# Manganese Removal from Liquid Nickel by Hydrogen Plasma Arc Melting

**DOI:** 10.3390/ma12010033

**Published:** 2018-12-22

**Authors:** Xiliang Guo, Jianbo Yu, Yuan Hou, Yujia Zhang, Jiang Wang, Xia Li, Hanlin Liao, Zhongming Ren

**Affiliations:** 1 State Key Laboratory of Advanced Special Steel, Shanghai University, Shanghai 200444, China; xlguo@shu.edu.cn (X.G.); houyuan@i.shu.edu.cn (Y.H.); yujzhang@shu.edu.cn (Y.Z.); jiangwang@i.shu.edu.cn (J.W.); xiali@shu.edu.cn (X.L.); 2 School of Material Science and Engineering, Shanghai University, Shanghai 200444, China; 3 ICB UMR 6303, CNRS, University Bourgogne Franche-Comté, UTBM, F-90010 Belfort, France; hanlin.liao@utbm.fr

**Keywords:** manganese removal, nickel melt, hydrogen plasma arc melting, kinetic, thermodynamic

## Abstract

In this work, the removal of manganese from nickel melts by Ar and (10%, 20% and 40%) H_2_ plasma arc melting under various pressures (0.01–0.02, 0.04–0.05 and 0.09–0.1 MPa) was investigated experimentally. The results show that only a slight reduction in the manganese content is obtained by Ar plasma arc melting (PAM). By contrast, the manganese content of liquid nickel decreases noticeably upon the addition of hydrogen to plasma gas, and the rate of manganese removal increases with increasing hydrogen volume fraction. In addition, the reduction in the pressure enhances the efficiency of manganese removal from liquid nickel by hydrogen plasma arc melting (HPAM). The process of manganese removal by HPAM was found to obey a first-order rate law. From kinetic analysis, the rate of reduction in the manganese content increases proportionally to the 0.73–0.75th power of the hydrogen volume fraction in the plasma gas. However, the rate of the manganese content reduction increases proportionally to approximately 0.88th power of %H_2_ in the plasma gas for the initial manganese content of 0.89 mass%, which is slightly higher than that for the initial manganese concentration of 0.45 mass%. Thermodynamic analysis indicates that the volatilization of manganese benefits from negative pressure and the presence of active hydrogen atoms that act as the transfer media of the metal vapor in the gas boundary layer.

## 1. Introduction

The demand for high-purity nickel has been increasing in recent years due to its excellent mechanical properties. Nickel is a key element in high-temperature stress-resistant and corrosion-resistant superalloys used in the aerospace industry [1]. In addition, pure nickel has also been applied in electrodeposited Ni-Co coating materials [2], biomaterials [3], and low-expansion alloys [4] and is a potential tool material for the thixoforming of steels [5]. High-purity nickel is also a key material in the production of hydrogen-generation catalysts and other chemicals [6]. Obviously, the impurities in the metal are harmful to its properties. Therefore, removal of impurities from nickel has always been a focus of research.

To date, non-metallic impurities have been eliminated by various agents, such as Si, Hf, Y, Al, Mg, and Zr [7,8,9,10,11,12]. The metallic impurities have been easily removed by electric beam melting (EBM) through its conditions of high vacuum and high temperature during melting [13]. However, its significant weight loss and high cost have hindered the wide use of EBM. Recently, hydrogen plasma arc melting (HPAM) was developed for refining active metals such as Hf, Cr, Zr, Nb, Ti, Tb, and Gd [14,15,16,17,18,19,20,21,22]. It was found that hydrogen in the plasma could increase the efficiency of metallic impurity removal. However, few investigations have been done on the purification of nickel by HPAM.

In this work, the effect of the hydrogen content in the plasma gas, melting pressure, and initial manganese content on the removal of a typical metallic impurity manganese by HPAM has been studied systematically. The kinetics and thermodynamics of this process were examined to investigate the mechanism of the manganese removal by HPAM.

## 2. Experimental Sections

**Figure 1:**
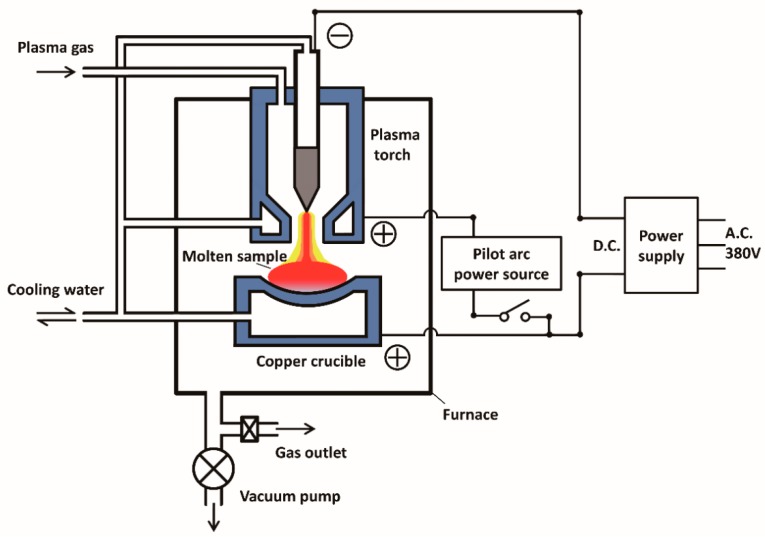
Schematic diagram of plasma arc furnace.

As shown in Figure 1, the plasma arc furnace with a transferred arc type plasma torch (Zhen Bang Areospace Precision Machinery Co. Ltd, Beijing, China) was used in the present work. A stable non-transfer arc was generated by the pilot arc power source. The D.C. power (transformed from a 3-phase A.C. power) was applied to obtain the transfer arc. The plasma arc power used for melting was 6 kW. A water-cooled Cu crucible was used as the anode. The cathode was made from tungsten and was placed in the plasma torch. The raw nickel ingot (containing approximately 0.45 mass% and 0.89 mass% Mn, approximately 25 g) was loaded on the water-cooled Cu crucible of 40 mm in diameter and 6 mm in depth. To eliminate the residual gas, the plasma arc furnace was evacuated to 6 × 10^−3^ Pa and flushed with high-purity Ar gas. The high-purity Ar and H_2_ plasma gas were mixed and introduced into the plasma torch. The flow rate of the plasma gas was constantly 5 L/min. The volume fraction of H_2_ in the plasma gas was 10%, 20% and 40%, respectively. For uniform refinement, the sample was melted again after being turned over. The total melting times for each specimen were 30, 60 and 120 min, respectively. The melting process was conducted under the pressures of 0.01–0.02, 0.04–0.05, and 0.09–0.1 MPa, respectively. The amount of manganese was determined using an optical emission spectrometer (OES, SPECTRO, Kleve, Germany). All specimens were grinded and polished before analyzed by OES.

## 3. Results and discussion

**Figure 2:**
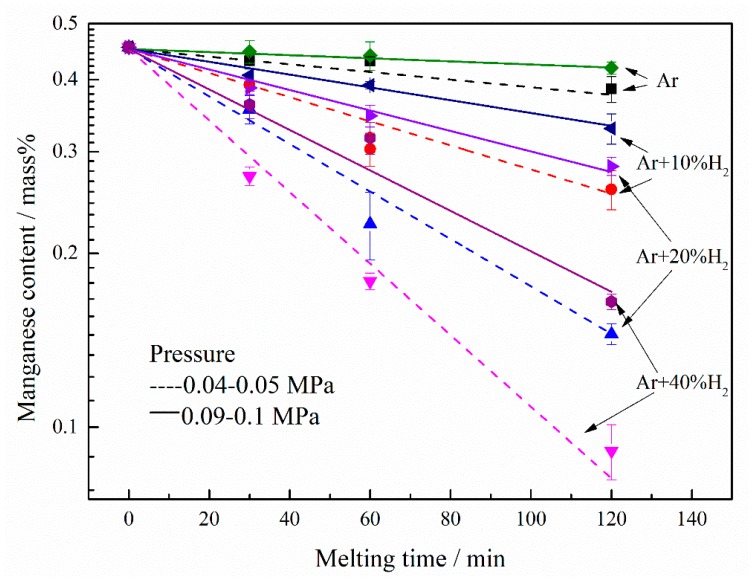
Change in the manganese content of liquid nickel during HPAM at 0.04–0.05 and 0.09–0.1 MPa.

The changes in the manganese concentration in the liquid nickel by argon plasma arc melting (PAM) and hydrogen plasma arc melting (HPAM) under pressure of 0.04–0.05 and 0.09–0.1 MPa are illustrated in Figure 2 as a function of melting time. For the pressure of 0.09–0.1 MPa, the manganese content decreased slowly after PAM. However, the manganese concentration decreased obviously with the addition of hydrogen in the plasma gas, in Figure 2. After 120 min of HPAM with addition of 10%, 20% and 40%H_2_ in plasma gas, the manganese content of decrease from about 0.45% to 0.32%, 0.28%, and 0.16%, respectively. On the other hand, a similar phenomenon about the manganese concentration reduction after HPAM was found under the pressure of 0.04–0.05 MPa. A slight reduction of manganese content was observed when the plasma gas consisted of argon only. The manganese concentration decreased significantly by the adding of hydrogen in the plasma gas. For the proportion of hydrogen at 10%, 20%, and 40%, after 120 min melting of HPAM, the manganese content of decrease from about 0.45% to 0.25%, 0.14%, and 0.09%, respectively. Moreover, the decrease rate in manganese concentration increased with the hydrogen proportion in the plasma gas.

Furthermore, the relationship between the logarithm of the manganese content and the melting time is almost linear, indicating that the process of manganese removal from liquid nickel by HPAM obeys a first-order rate law. There is a slight scattering around the straight lines. However, the data point with error bar was almost on the line. The apparent manganese reduction rate constant $k_{Mn}$ and the standard error of the lines of best fitting were listed in Table 1. It indicated that the standard error is much smaller than the manganese reduction rate constant. Therefore, the standard error can be ignored. It suggests that minor composition segregation occurs in the samples. It is clearly observed that the slope of the solid line is smaller than that of the dashed line, which means that the rate of manganese removal under the pressure of 0.04–0.05 MPa is larger than that under the pressure of 0.09–0.1 MPa.

The process of manganese removal from the liquid nickel can be expressed by Equation (1), where [%*Mn*] and $k_{Mn}$ are the manganese concentration in mass% and the apparent manganese reduction rate constant, respectively. Integration of Equation (1) gives Equation (2), in which [%*Mn*]_0_ and [%*Mn*]*_t_* are the manganese concentrations at *t* = 0 and *t* = t, respectively. Based on Equation (2), the values of $k_{Mn}$ at the respective hydrogen contents in the plasma gas can be derived from the slopes of the straight lines in Figure 2. On the one hand, under the pressure of 0.09–0.1 MPa, for the hydrogen volume fraction of 10%, 20% and 40%, the apparent manganese reduction rate constants were 2.74 × 10^−3^, 4.08 × 10^−3^ and 7.74 × 10^−3^ min^−1^, respectively. On the other hand, under the pressure of 0.04–0.05 MPa, the apparent manganese reduction rate constants were 4.97 × 10^−3^, 9.56 × 10^−3^, and 1.37 × 10^−2^ min^−1^, corresponding to the hydrogen fractions of 10%, 20%, and 40%, respectively.

(1) $$-d[\%Mn]/dt=K_{Mn}\cdot [\%Mn]$$

(2) $$\mathrm{lg}{\left[\%Mn\right]}_{0}-\mathrm{lg}{\left[\%Mn\right]}_{t}=K_{mn}\cdot t/2.303$$

**Figure 3:**
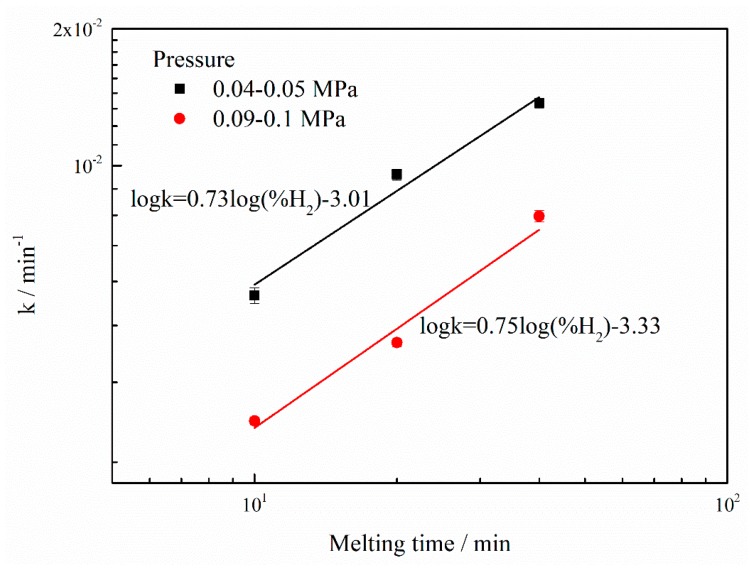
Dependence of the apparent manganese reduction rate constant ($k_{Mn}$) on the hydrogen volume proportion (%*H_2_*).

As described in Figure 3, the apparent reduction rate constant $k_{Mn}$ is plotted against the hydrogen volume fraction %*H_2_* on the log-log scale. It is found that a good linear relationship exists between $k_{Mn}$ and %*H_2_*. According to the calculated results, for the pressure of 0.04–0.05 MPa, the slope of the plot of $k_{Mn}$ against %*H_2_* is approximately 0.73, as expressed in Equations (3) and (3a). Equation (4) is obtained by substituting Equation (3a) into Equation (1). Equation (4) indicates that the reduction rate of manganese increases linearly to approximately 0.73th power of %*H_2_* in the plasma gas.

(3) $$K_{Mn}\propto {\left(\%H_{2}\right)}^{0.73}$$

(3a) $$K_{Mn}=K_{Mn}{}^{\prime }{\left(\%H_{2}\right)}^{0.73}$$

(4) $$-d[\%Mn]/dt=K_{mn}{}^{\prime }{\left(\%H_{2}\right)}^{0.73}\cdot [\%Mn]$$

On the other hand, the reduction rate under the pressure of 0.09–0.1 MPa is observed to increase proportionally to approximately 0.75th power of the hydrogen fraction. This suggests that there is no significant difference between the slopes in Figure 3 for the pressures of 0.04–0.05 and 0.09–0.1 MPa.

**Figure 4:**
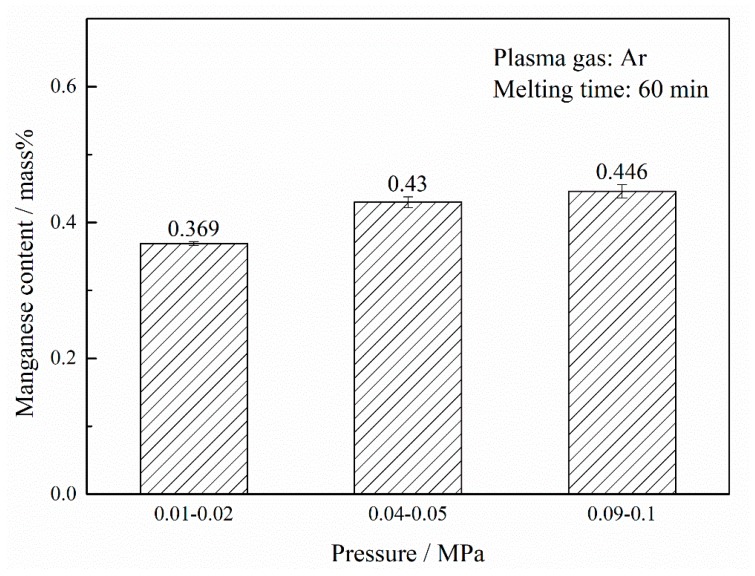
Manganese content after PAM under different pressures.

According to the $k_{Mn}$ values under different pressures and various hydrogen fractions in the plasma gas, the manganese removal rate increases with the augment of hydrogen content in the plasma gas. In addition, the $k_{Mn}$ values under the lower pressure of 0.04–0.05 MPa were higher than those for the pressure 0.09–0.1 MPa. This indicates that dropping pressure is a beneficial method for removing manganese from the nickel melt. Therefore, under the pressure of 0.01–0.02 MPa, the experiments for manganese removal from liquid nickel by PAM and HPAM were performed. However, it was found that the transferred plasma arc was unstable when hydrogen was added to the plasma gas at the pressure of 0.01–0.02 MPa. The results after melting for 60 min by PAM are shown in Figure 4, and it is observed that the manganese concentration clearly decreases in pressure.

**Figure 5:**
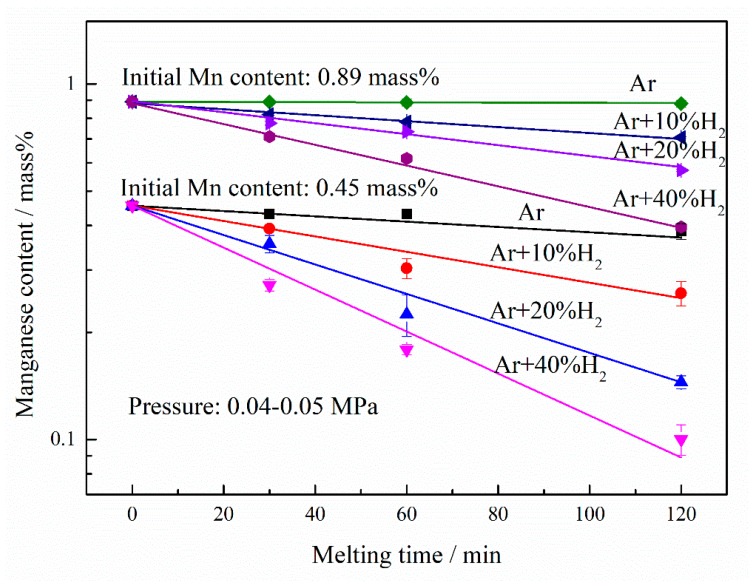
Change in the manganese content of liquid nickel during HPAM with different initial manganese contents (0.89 mass% and 0.45 mass%).

**Figure 6:**
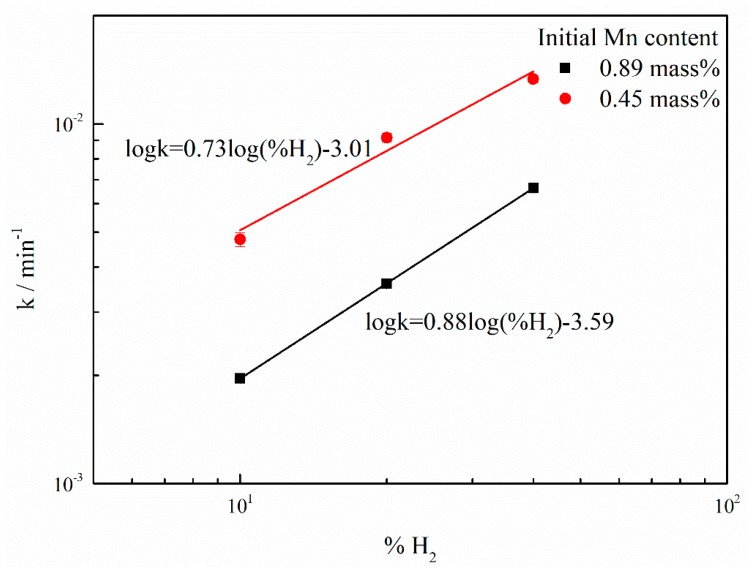
Dependence of the apparent manganese content reduction rate constant ($k_{Mn}$) on the hydrogen volume fraction (%*H_2_*) for different initial manganese contents (0.89 mass% and 0.45 mass%).

With the different initial manganese concentrations, the changes in the manganese content in the nickel melt during PAM and HPAM as a function of the melting time were illustrated in Figure 5. Be the same as shown in Figure 1, the manganese content declined slightly after PAM and clearly decreased with the addition of hydrogen to the plasma gas. Furthermore, the rates of manganese reduction were calculated, according to Equation (2) and based on the slopes of the fitting lines in Figure 5. For the initial manganese content of 0.89 mass% and the volume fraction of hydrogen of 10%, 20% and 40%, the apparent manganese content reduction rate constants were 1.96 × 10^−3^, 3.59 × 10^−3^ and 6.63 × 10^−3^ min^−1^, respectively. On the other hand, for the initial manganese concentration of 0.45 mass%, the apparent manganese content reduction rate constants were 4.97 × 10^−3^, 9.56 × 10^−3^ and 1.37 × 10^−2^ min^−1^, corresponding to the hydrogen fraction of 10%, 20% and 40%, respectively. This shows that the manganese content reduction rate increases with the decrease in the initial manganese content. Figure 6 shows the apparent reduction rate constant $k_{Mn}$, and $k_{Mn}$ plotted against the hydrogen volume fraction %*H_2_* on the log-log scale. It is observed that there is a good linear relationship between $k_{Mn}$ and %*H_2_*. Under the pressure of 0.04–0.05 MPa, for the initial manganese content of 0.45 mass%, the slope of the plot of $k_{Mn}$ versus %*H_2_* is approximately 0.73. On the other hand, the slope of $k_{Mn}$ against %*H_2_* is about 0.88 for the manganese content of 0.89 mass%. This suggests that the manganese content reduction rate increases proportionally to 0.73th power of the %*H_2_* in the plasma gas for the initial manganese content of 0.45%, which is a slightly lower than that for the initial manganese concentration of 0.89%.

**Figure 7:**
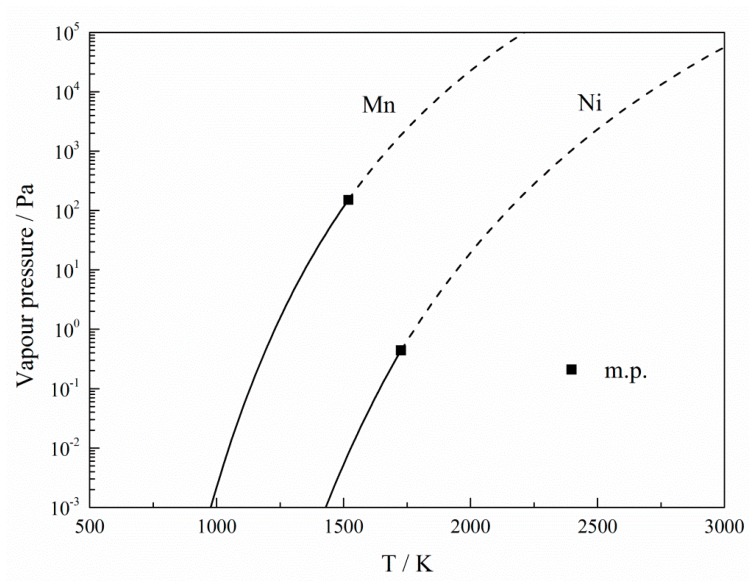
Vapor pressures of manganese impurity and nickel metal as a function of temperature.

Based on the above mentioned results, HPAM was confirmed to be an efficient method for removing metallic impurities. The beneficial effect for manganese removal from the nickel melt is closely related to the vapor pressures of manganese and nickel. The thermochemical equations of manganese and nickel are described in Equations (5)–(7) [23]. According to the equations, the vapor pressures of manganese and nickel as a function of temperature are shown in Figure 7. It is observed that manganese has a higher vapor pressure than nickel, which means that manganese evaporates much easier than nickel. This suggests that the manganese impurity can be readily removed from liquid nickel during HPAM. Compared to the atmospheric pressure conditions, the vaporization of manganese is enhanced under low pressure, because of the higher activity coefficient of manganese in the nickel melt at negative pressure [18,24]. Therefore, a low melting pressure is an important factor for removing manganese from liquid nickel.

(5) Mn:lgP=5.006+12.805−15097/T−1.7896lgT(298K−m.p.)

(6) Ni:lgP=5.006+10.557−22606/T−0.8717lgT(298K−m.p.)

(7) lgP=5.006+6.666−20765/T(m.p.−2150K)

**Figure 8:**
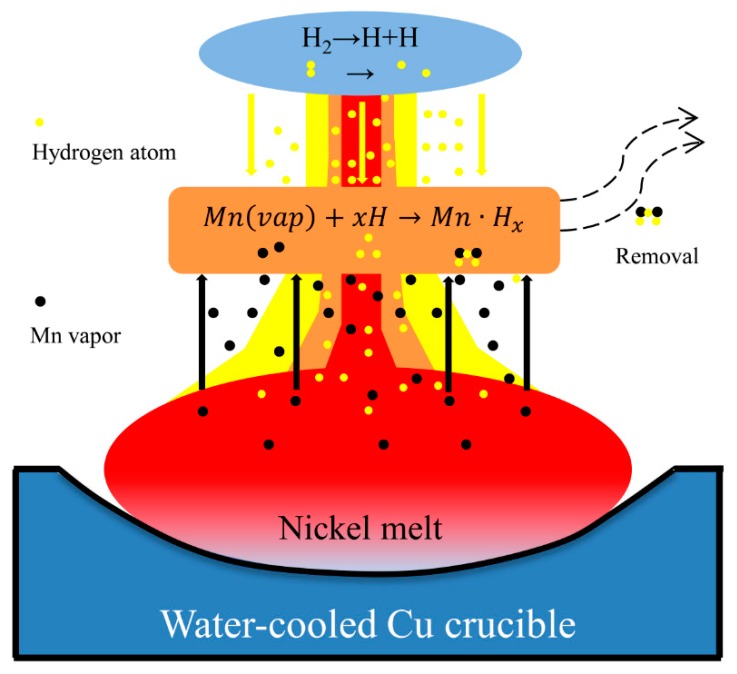
Schematic diagram of manganese removal from nickel melt during HPAM.

Addition of hydrogen to plasma gas is another important way to enhance the removal of manganese. Figure 8 shows a schematic diagram of the manganese removal process during HPAM, and the process can be expressed by Equation (8). As shown in Figure 8, the evaporated manganese ($Mn_{vap}$) reacts with active hydrogen atoms to form manganese hydrides ($Mn\cdot H_{x}$) that would dissociate to manganese and hydrogen at low temperatures. In other words, the metal vapor within the gaseous boundary was enhanced by the dynamic interaction between the hydrogen atoms and the metal vapor [25]. Therefore, the manganese content reduction rate can be noticeably increased even under atmospheric pressure when hydrogen was added to plasma gas. Approximately 95% of the hydrogen molecules dissociated to hydrogen atoms under the high electric field from the transferred arc power at 5000 K [26]. That is to say, the hydrogen molecules dissociate to atoms in the plasma arc when hydrogen is added to the plasma gas. Due to the high thermal conductivity of the activated hydrogen atoms, the heat capacity and heat conductivity of the plasma are enhanced by the adding hydrogen compared to the case when the plasma gas consisted of argon only. As a result, the temperature of the nickel melt increases when the active hydrogen atoms were dissolved in the liquid nickel. It is observed from Figure 8 that the increasing temperature can improve the vapor pressure of manganese. This indicates that elevating the temperature by adding hydrogen to plasma gas enhances the removal of manganese from the nickel melt. Furthermore, the reaction described in Equation (8) is also accelerated at elevating temperature.

(8) $$Mn(vap)+xH\to Mn\cdot H_{x}\to Mn+x/2H_{2}$$

## 4. Conclusions

In the present study, removal of manganese from nickel melt by HPAM is studied systematically. The concentration of manganese in liquid nickel decreases slowly with melting time when the plasma gas consists of argon only. However, the manganese content decreases noticeably upon the addition of hydrogen to the plasma gas. Moreover, the rate of manganese removal increases with the reduction in the pressure. Kinetic analyses suggest that the process of manganese removal from liquid nickel by HPAM obeys the first-order kinetics rate law. The manganese content reduction rate increases proportionally with approximately 0.73–0.75th powers of the hydrogen volume fraction in the plasma gas. The hydrogen volume fraction exponent increases to 0.88th when the initial manganese content increases from 0.45 mass% to 0.89 mass%. The thermodynamic results indicate that the low pressure and high temperature of the melt promote the volatilization of manganese from liquid nickel. The dissociation of the hydrogen molecules to active hydrogen atoms improves the temperature of the melt due to the high heat conductivity of the active hydrogen atoms.

## Figures and Tables

**Table 1:** The manganese reduction rate constant and its corresponding standard error.

Plasma gas	$k_{Mn}$ under 0.09–0.1 MPa	Standard Error	$k_{Mn}$ under 0.04–0.05 MPa	Standard Error
Ar	6.24 × 10^−4^	2.0 × 10^−5^	1.20 × 10^−3^	5.4 × 10^−5^
Ar-10%H_2_	2.74 × 10^−3^	5.6 × 10^−5^	4.97 × 10^−3^	2.1 × 10^−4^
Ar-20%H_2_	4.08 × 10^−3^	8.3 × 10^−5^	9.56 × 10^−3^	2.7 × 10^−4^
Ar-40%H_2_	7.74 × 10^−3^	2.2 × 10^−4^	1.37 × 10^−2^	3.3 × 10^−4^
